# A three-year cohort study of the relationships between coping, job stress and burnout after a counselling intervention for help-seeking physicians

**DOI:** 10.1186/1471-2458-10-213

**Published:** 2010-04-27

**Authors:** Karin E Isaksson Ro, Reidar Tyssen, Asle Hoffart, Harold Sexton, Olaf G Aasland, Tore Gude

**Affiliations:** 1 The Research Institute, Modum Bad, NO-3370 Vikersund, Norway; 2 Department of Behavioural Sciences in Medicine, Institute of Basic Medical Sciences, Faculty of Medicine, University of Oslo, Boks 1111 Blindern, NO-0317 Oslo, Norway; 3 Department of Psychology, University of Oslo, Norway; 4 Research Institute of the Norwegian Medical Association, Postbox 1152 Sentrum, NO-0107 Oslo, Norway; 5 Department of Health Management and Health Economics, Institute of Health and Society, University of Oslo, Norway

## Abstract

**Background:**

Knowledge about important factors in reduction of burnout is needed, but there is a dearth of burnout intervention program studies and their effects among physicians. The present three-year follow-up study aimed to investigate the roles of coping strategies, job stress and personality traits in burnout reduction after a counselling intervention for distressed physicians.

**Methods:**

227 physicians who attended a counselling intervention for burnout at the Resource Centre Villa Sana, Norway in 2003-2005, were followed with self-report assessments at baseline, one-year, and three-year follow-up. Main outcome measures were emotional exhaustion (one dimension of burnout), job stress, coping strategies and neuroticism. Changes in these measures were analyzed with repeated measures ANOVA. Temporal relationships between changes were examined using structural modelling with cross-lagged and synchronous panel models.

**Results:**

184 physicians (81%, 83 men, 101 women) completed the three-year follow-up assessment. Significantly reduced levels of emotional exhaustion, job stress, and emotion-focused coping strategies from baseline to one year after the intervention, were maintained at three-year follow-up.

Panel modelling indicated that changes in emotion-focused coping (z = 4.05, p < 0.001) and job stress (z = 3.16, p < 0.01) preceded changes in emotional exhaustion from baseline to three-year follow-up. A similar pattern was found from baseline to one-year follow-up.

**Conclusion:**

A sequential relationship indicated that reduction in emotion-focused coping and in job stress preceded reduction in emotional exhaustion. As a consequence, coping strategies and job stress could be important foci in intervention programs that aim to reduce or prevent burnout in help-seeking physicians.

## Background

The prevalence and predictors for the development of burnout in the medical profession have been explored to some extent [1-4], but we need follow-up studies after interventions for burnout to examine the role of coping strategies, job stress and personality traits for the reduction of burnout.

Coping strategies have been defined as strategies used to reduce the possible harm of an event that is considered potentially dangerous to the person's psychological well-being [5], and they are usually grouped into (1) active, problem-focused and (2) emotion-focused or emotional ways of coping. Wishful thinking, an emotion-focused way of coping, has been found to be associated with depression in medical students [6], and has predicted need of mental health treatment in young doctors [7]. In cross-sectional studies, emotion-focused coping has also been associated with job stress and burnout among physicians [8,9] and with post traumatic stress disorder among Israeli physicians [10]. The use of active coping strategies has been found to increase with stress from malpractice litigations [11] and with work-related stress due to racism [12]. Therapeutic interventions, with the potential to change unfavourable coping strategies, have been found in patient populations faced with chronic stress [13]. It is therefore important to study whether interventions such as counselling or psychotherapy could also influence changes in coping strategies among distressed physicians, and whether such changes are related to reduction in emotional exhaustion.

Theoretically, work load precedes work stress, which then may lead to burnout. This process is influenced by both individual characteristics (e.g. personality and ways of coping) and organizational factors (e.g. specialist status). It could thus be expected that reduction in job stress could have an impact on reduction in burnout [14]. In cross-sectional samples an association between job stress (and two of its sub-dimensions, time pressure and work-home interface stress) and emotional exhaustion has been found among physicians [2,4]. In longitudinal normative samples of physicians, however, only reciprocal and no clear one-way directional relationships between job stress and emotional exhaustion have been found [15,16]. A few studies from the U.S. have found an association between number of work hours (one component of work load) and burnout among physicians [17-19], while other studies (mainly European) have failed to do so [4,20-22]. This may be due to longer work hours in the U.S. A reduction in number of work hours/week, however, has been shown to be associated with reduction in emotional exhaustion, not only among U.S. residents [23-25], but also in a cohort of Norwegian physicians [26]. Thus, factors that have not been clearly documented as predictors of burnout development may nevertheless be important facilitating its remission.

The personality trait most consistently associated with emotional exhaustion is neuroticism [27]. In a cross-sectional study of physicians this trait had 31% shared variance with emotional exhaustion [8], and in long-term prospective studies neuroticism has been found to predict emotional exhaustion in physicians [3,28]. The role of neuroticism is thus important to study in relation to interventions for burnout.

There is little documentation of interventions to reduce or prevent burnout among physicians. Reduction in emotional exhaustion, often described as the primary dimension of burnout [27,29], has been found six weeks after a stress management workshop for residents [30]. Following a general work hour limitation for residents in the United States in 2003, several studies found reduced rates of emotional exhaustion six months to a year later [23-25,31], whereas one study indicated that no significant reduction was found after two years in a cohort of residents in orthopaedic surgery [17]. In a follow-up after a preventive intervention for health care workers, including physicians, initial reduction of emotional exhaustion was found, but levels increased again through two years of follow-up in the group not receiving further interventions [32]. These results emphasize the need for follow-up studies over more than one to two years, in order to study the long-term course of burnout, as well as factors important for reduction and stabilization of burnout levels after counselling or therapeutic interventions.

A counselling intervention program offered to physicians at a Resource Centre for Health Personnel, Villa Sana in Norway, emphasized two main intervention areas. One was mapping and discussion of the physicians' current life situation, with an emphasis on job stress and its reduction - often job stress related to the work-home interface. The other was identifying and challenging the coping strategies being used by the physicians [26]. Baseline data from this program indicated that the intervention programs reached physicians in need of help [33], and data from one-year follow-up indicated a substantial reduction of emotional exhaustion [26]. Data on coping strategies in this cohort have not been reported previously.

On this basis, we examined the following questions at three-year follow-up with data from the same cohort.

1. How will emotional exhaustion, job stress, coping strategies and neuroticism change from baseline to three-year follow-up?

2. What is the sequential relationship between changes in emotional exhaustion and changes in job stress, ways of coping and neuroticism from baseline to follow-up?

3. Will ways of coping be affected by therapy undertaken after the intervention?

## Methods

### Study design and sample

**Figure 1:**
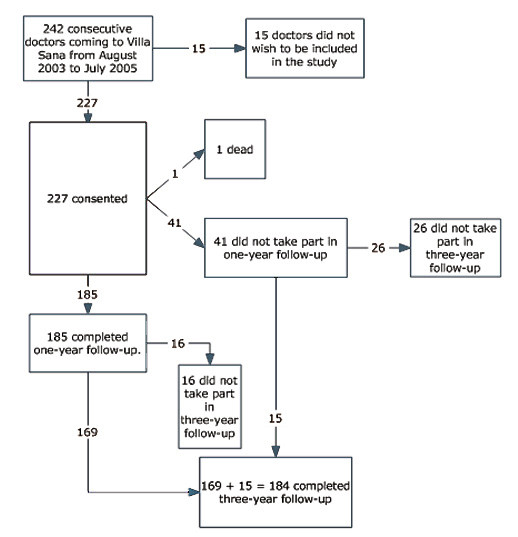
**Flow chart over participation in the study**.

The consecutively participating physicians in a counselling intervention at The Resource Centre for Health Personnel in Norway, from August 2003 through July 2005, were eligible for inclusion in the study. Participants signed an informed written consent. The cohort comprised 227 physicians at baseline (94% of 242 eligible) (see Figure 1). Self-report instruments were completed before the intervention (baseline) and were mailed to participants approximately one and three years after the intervention (two reminders sent). Three-year follow-up was completed 36.9 months (SD 1.9, range 34-44.5) after baseline.

### Intervention

The Resource Centre is available for all Norwegian physicians. It is funded by the Norwegian Medical Association and is located at a psychiatric facility, Modum Bad.

The interventions are based on an integrative approach incorporating psychodynamic, cognitive and educational theories [26].

Physicians chose to participate in one of two different interventions. The first was a single day, six to seven hour counselling session for one physician with a psychiatrist or a specialist in occupational medicine (MD). A "non-treatment" setting without medical records and with absolute confidentiality was ensured. After being invited to describe his or her situation the physician was asked to map both work-related and private contextual factors contributing to stress. Coping strategies, often related to sources of identity, self-esteem and self-reliance in the individual, were identified, acknowledged, and challenged. The physician's present needs in both a short and a longer perspective were identified, and it was usually recommended that the doctor actively should deal with these needs (for example by stress reduction or obtaining treatment, such as psychotherapy).

The second type of intervention was a five day, group based course for eight participants, boarding at the Centre, and led by one of the same counsellors in collaboration with an occupational therapist. Daily lectures, group discussions, and physical activity were offered as well as an individual counselling session during the week. The intervention is described in more detail elsewhere [26,33].

### Variables

#### Burnout

In this study we used the subscale emotional exhaustion (10 items, Cronbach's α = .92) of Maslach's Burnout Inventory [34]. As in previous studies of Norwegian physicians, a five-point scale scoring perceived fit (1-does not fit, 5-fits very well), with reference to the last two weeks at work, was used to score the MBI [26,29], contrary to the seven-point scale scoring frequency that is used in most studies internationally. The seven-point scale has been criticized for having categories that are not mutually exclusive [35].

#### Personality

Eysenck's abbreviated personality questionnaire with six items for neuroticism explaining 82% variance of the original scale was used (α = .71) [36]. Items were scored dichotomously (1-yes or 0-no), and a sum score from 1-6 was obtained, in which higher scores designate more neuroticism.

#### Perceived job stress

17 items from a modified version of the Cooper Job Stress Questionnaire, used in the Norwegian student/doctor cohort, were selected using Principal Component Analysis. In addition nine clinically prompted items from the questionnaire were included, as previously described [26]. Job stress with 26 items (α = .92), as reported in this paper, consists of three subscales - emotional stress (10 items, α = .85), social stress, including work-home interface stress and time stress (10 items, α = .83) and fear of litigation which also covers fear of complaints or criticism (6 items, α = .86). The correlations between the subscales ranged from .48 to .64. Scores were given on a five-point scale (1 = no stress, 5 = very much stress), with reference to the two last weeks at work.

#### Coping strategies

Eighteen of 42 items in Vitaliano and colleagues' Ways of Coping Checklist were selected by a Principal Component Analysis of data from the Norwegian student/doctor cohort [6,7]. Vitaliano defines two main dimensions of coping strategies - the more adaptive ways of coping and the potentially maladaptive ways of coping [6]. Among the adaptive strategies two subscales are "problem-focused coping" and "seeking social support." In this study they are described with four and five items respectively, together designated as "active coping strategies" (α = .81). Among the potentially maladaptive strategies two subscales are "wishful thinking" and "blaming self." In this study they are described with seven and two items respectively, together designated as "emotion-focused coping strategies" (α = .82). (For a list of items, see Additional file 1). Scores were given on a five-point scale (1 = does not fit at all and 5 = fits completely).

#### Demographic data

Gender, age, marital status, having children less than 16 years of age (dichotomous variable).

#### Specialist status

Categorized into internal medical specialties, surgical specialties, psychiatric specialties, general practice (in Norway general practice or family medicine is an approved specialty, and about half of the general practitioners are specialists), public health and laboratory medicine, non-specialist (usually specialists in training) [33].

#### Work hours

Sum of hours per week used in direct patient contact, meetings, paperwork, on the telephone etc., research, and "other work activities" [26].

#### Psychotherapy

Attending psychotherapy during the first year after baseline intervention (0-no and 1-yes).

#### Sick leave

Number of weeks on full time/part time sick leave/rehabilitation/disability during the preceding year and current sick leave.

### Statistics

Continuous, repeated parameters were tested with repeated measures ANOVA (repeated contrast) with time (baseline, one-year and three-year), and with interactions between time and psychotherapy. Variables were normally distributed. The condition of sphericity was examined with Mauchly's test. In case of violation of the assumption of sphericity, degrees of freedom were corrected using Greenhouse-Geisser estimates of sphericity (ε = .91).

Two repeated measures in the same cohort were tested with paired t-tests for parametric data, Wilcoxon's rank test for continuous, non-parametric data, and McNemar's test for dichotomous variables. T-tests and Chi-square tests were used respectively for comparison between different groups.

Effect sizes using pooled SD were calculated according to the method of Cohen (the mean difference/sum of standard deviation for the two measures/2), defining values of <0.20 as indicating no effect, 0.20-0.49 indicating small and 0.50-0.79 indicating moderate effect [37].

**Figure 2:**
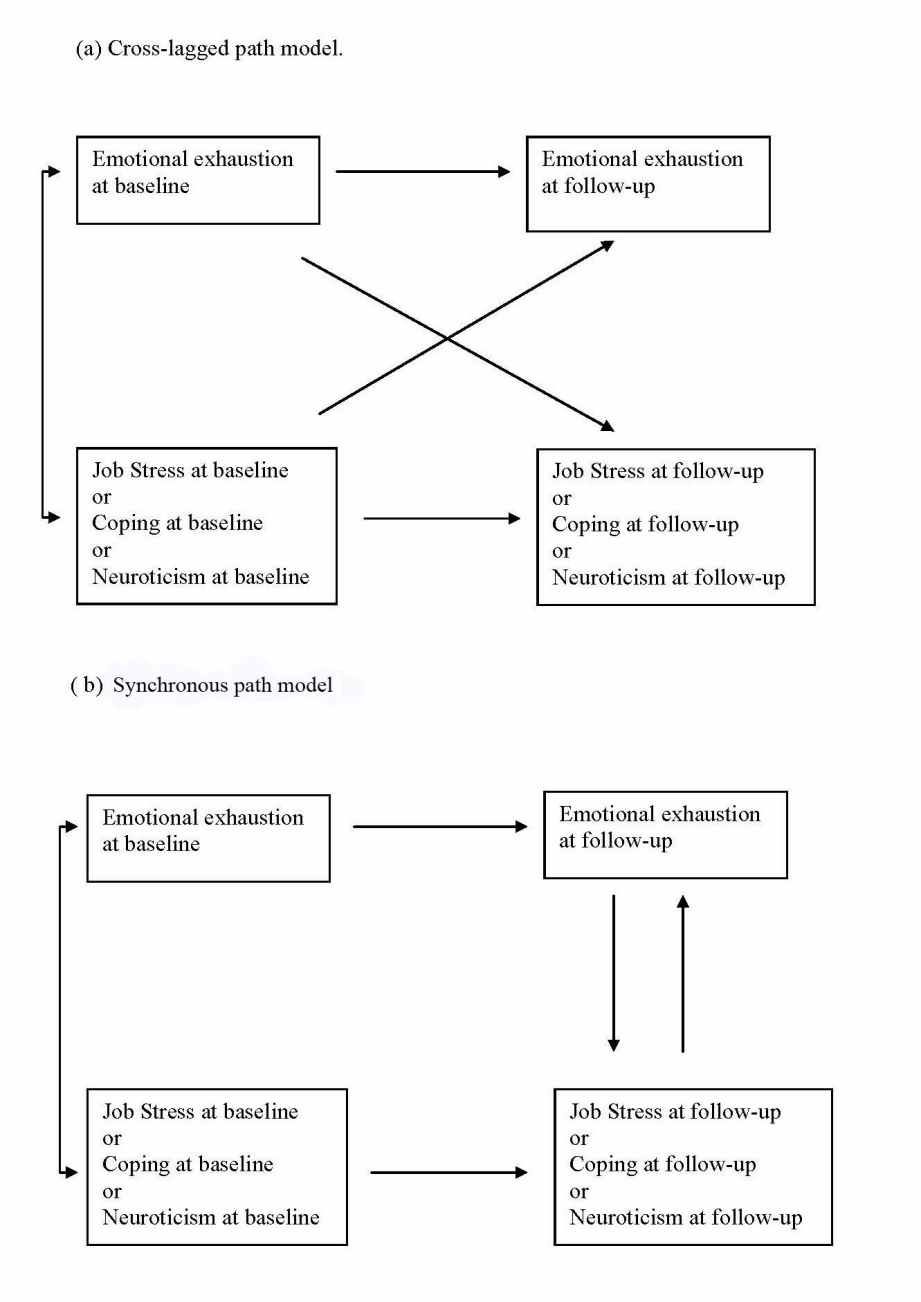
**Cross-lagged and synchronous/co-temporal panel models**.

The sequential relationships between change in emotional exhaustion relative to changes in job stress, coping and neuroticism respectively were examined using the structural modelling program EQS 6.1, beta version, in a series of cross-lagged and synchronous panel models. Cross-lagged models examine if the baseline value of one parameter influences change in the other parameter. The synchronous or co-temporal model examines if change in one parameter appears to influence change in the other parameter [[38], pp 22-37]. (See Figure 2). As the time span from the intervention to follow-up (to both one-year and three-years) was relatively long, we would expect that synchronous or co-temporal panel models, rather than cross-lagged, would be likely to detect temporal relationships that might be present between these variables [[38], p32]. Cross-lagged paths were examined first. The synchronous relationships were studied if the cross-lagged paths had an inadequate model fit or did not show significant cross-lagged relationships (Figure 2).

The variables were allowed to freely correlate at baseline. The model fit criterion of the confirmatory fit index, CFI, is a frequently used measure of the adequacy with which the structural model represents the observed data and was used to determine the adequacy, or fit, of the model. A comparative fit index (CFI) >= 0.95 in combination with the standardized root mean squared residual (SRMR) <= 0.08 (which is independent of sample size) were used to determine model fit [39]. A critical ratio (parameter/standard error) of 1.96 or greater was used to determine whether or not a path was significant at the 0.05-level.

The sample size was adequate for maximum likelihood estimation of models with a small number of parameters to be estimated, but not for the use of latent variables [39]. The relationships were studied from baseline to both one- and three-year follow-up. Cronbach's α-values from 0.71 - 0.92 indicate satisfactory internal consistency of the variables. The lack of excessive kurtosis or skewness in the variables indicated a sufficiently normal distribution of the data. The correlations at baseline, one-year and three-year follow-up were between emotional exhaustion and (i) job stress 0.70, 0.64 and 0.67 (ii) active coping -0.23, -0.21 and -0.23 (iii) emotion-focused coping 0.47, 0.59 and 0.62 (iv) neuroticism 0.53, 0.61 and 0.55 with significance levels ranging between <0.01 and <0.001.

The level of significance was set to p < 0.05 in general. To avoid type I-error due to number of tests a statistical correction was performed in relation to the ANOVAs and to the panel modelling. This correction implied that for the four overall ANOVAs a p-value of <0.05/4 = 0.0125 should be required. The overall ANOVAs all had a significance of <0.001, indicating that the results were not incidental. For the panel models, involving fourteen tests, a p-value of <0.05/14 = 0.0036 should be required. This corresponds to a Critical ratio CR = parameter/standard error (distributed as z) of >2.93, indicating that, except for stress due to fear of litigation, the significant relationships found at three-year follow-up stay significant also after correction.

### Missing data

One or a few missing items in instruments measuring coping strategies and neuroticism were replaced by the mean score of completed items. The instruments measuring job stress and emotional exhaustion included items that were not relevant for all respondents due to differences in working conditions (not working directly with patients as in laboratory work, leadership, research). Mean score of relevant items for each individual was used.

Zero to four/184 (0-2.2%) of the instruments on burnout, job stress, coping and neuroticism at baseline, and 0-18/184 (0-9.8%) at three year follow-up were insufficiently completed. N was adjusted accordingly in the individual analyses.

### Ethics

Participants signed an informed, written consent. The study has been approved by the Data Inspectorate through the Norwegian Social Science Data Services. The Regional Ethical Research Committee in the South of Norway did not find special consent necessary for this study.

## Results

### Sample and attrition analysis

Three-year follow-up was completed by 184/227(81%), 83 men and 101 women. Data from all three assessment points (baseline, one-year, and three-year follow-up) were collected from 169/227 (74%) (see Figure 1). The included physicians were on average 46.9 years old at baseline. Several of the physicians who were training to become specialists at baseline had completed their training at follow-up (Table 1). The point prevalence and the number of weeks during the past year of full-time sick leave were significantly lower than at baseline (as also found at one-year follow-up [26]) (Table 1). The point prevalence of part-time sick leave, full- and part-time disability/rehabilitation/retirement benefits did not differ significantly from baseline (2-7/184; 1.1%-3.8%).

**Table 1:** Description of physicians at baseline and at three-year follow-up after a counselling intervention.

	Baseline	Three-year Follow up	Level of significance
	
	Mean (SD) or Number (%, 95% CI)	Mean (SD) or Number (%, 95% CI)	McNemar's test
**Age (years at baseline)** **n = 184**	46.8 (8.9)		

**Gender: Men/women** **n = 184**	83 (45%)/101 (55%)		

**Married/cohabiting** **n = 184**	152 (82.6, 77.1-88.1)	135 (73.4, 67.0-79.8)	p = 0.007**

**Have children aged <16 years**	90/184 (48.9, 41.7-56.1)	74/171 (40.2, 32.9-47.5)	p = 0.02*

**Specialty** **n = 184**			

Internal medicine	27 (14.7, 9.6 - 19.8)	34 (18.5, 12.9-24.1)	p = 0.02*

Surgery	30 (16.3, 11.0-21.6)	34 (18.5, 12.9-24.5)	p = 0.22

Psychiatry	16 (8.7, 4.6 - 12.8)	17 (9.2, 5.0 - 13.4)	p = 1.00

General practice	45 (24.5, 18.3 - 30.7)	50 (27.2, 20.8 - 33.6)	p = 0.18

Social and laboratory med.	16 (8.7, 4.6 - 12.8)	19 (10.3, 5.9 - 14.7)	p = 0.25

Non-specialist	50 (27.2, 20.8 - 33.6)	18 (9.8, 5.5 - 14.1)	p < 0.001***

Missing		12 (6.5, 2.9 - 10.1)	

**Proportion on full time sick leave at present n = 184**	60 (32.6%, 25.8 - 39.4))	10 (5.4%, 2.1 - 8.7)	p < 0.001***

			**t-test**

**Level of emotional exhaustion (1-5) n = 164**	3.0 (0.9)	2.4 (0.8)	t = 7.6 p < 0.001***

**Level of job stress (1-5) n = 173**	2.4 (0.7)	1.9 (0.6)	t = 8.2 p < 0.001***

**Level of emotion-focused coping (1-5) n = 183**	2.9 (0.8)	2.5 (0.8)	t = 7.0 p < 0.001***

**Level of neuroticism (1-6) n = 182**	2.6 (1.8)	2.0 (1.8)	t = 5.0 p < 0.001***

			**Wilcoxon rank test**

**Number of weeks on full time sick leave/disability/** **rehab benefits during the preceding year**	4.4 (7.9) (n = 172)	3.2 (9.8) (n = 178)	z = -3.1 p = 0.002**

**Work hours per week (h)**	43.2 (8.5) (n = 176)	39.6 (11.2) (n = 166)	z = -3.7 p < 0.001***

*p < 0.05, **p < 0.01, ***p < 0.001

The decreased number of work hours/week from baseline to one-year persisted at three-year follow-up (Table 1). Eighty-nine/184 (48.4%) reported attending psychotherapy during one-year follow-up. Base-line data for the whole cohort, including a comparison with Norwegian doctors in general, have been presented previously [33].

There were proportionally more women than men who completed the three-year follow-up (101/117, 86.3%, 95% CI 80.1 - 92.5 vs. 83/110, 75.5%, 95% CI 67.5-83.5, p = 0.04). Physicians not completing three-year follow-up (n = 43), compared with those completing three-year follow-up (n = 184), had significantly higher baseline levels of emotional exhaustion (3.33 SD 0.88 vs. 3.01 SD 0.94, p = 0.04), job stress (2.65 SD 0.74 vs. 2.39 SD 0.72, p = 0.03) and emotion-focused coping strategies (3.25 SD 0.63 vs. 2.90 SD 0.76, p = 0.006). There were no significant differences in age, the two other burnout dimensions, or active coping strategies.

### Changes from baseline to three year follow-up

There were significant changes from baseline to three-year follow-up in levels of emotional exhaustion, job stress, emotion-focused coping, and neuroticism (see Table 1). Effect sizes were moderate for changes in emotional exhaustion, job stress and emotion-focused coping (0.68, 0.72 and 0.49 respectively) and small for neuroticism (0.34).

**Figure 3:**
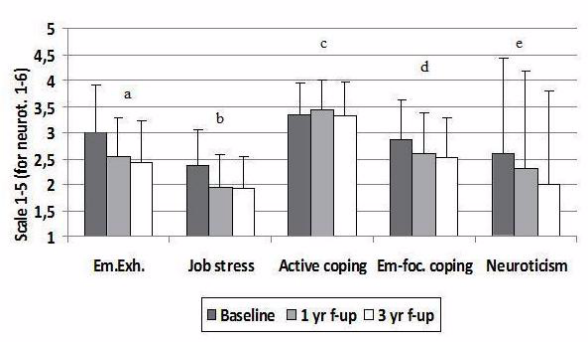
**Levels of emotional exhaustion, job stress, coping and neuroticism**. Repeated measures ANOVA for time (baseline, 1 yr, 3 yr) with contrasts for baseline-1 yr and 1 yr-3 yr. a. Overall ANOVA F(1.8, 267.0^†^) = 33.1*** n = 147. Baseline-1 yr F(1,146) = 39.3 ***. 1 yr-3 yr F(1,146) = 3.0. b. Overall ANOVA F(2,314) = 43.4*** n = 158. Baseline-1 yr F(1,157) = 66.6***. 1 yr-3 yr F(1,157) = 0.4. c. Overall ANOVA F(2,322) = 2.5 n = 162. d. Overall ANOVA F(2,320) = 24.5*** n = 161. Baseline-1 yr F(1,160) = 27.4***. 1 yr-3 yr F(1,160) = 1.9. e. Overall ANOVA F(2,322) = 10.1*** n = 162. Baseline-1 yr F(1,161) = 3.6. 1 yr-3 yr F(1,161) = 6.7**. *p < 0.05, **p < 0.01, ***p < 0.001. ^† ^- degrees of freedom corrected with Greenhouse-Geisser estimates, since Mauchly's test showed a violation of the assumption of sphericity.

Figure 3 shows overall changes and changes from baseline to one year and from one to three years (repeated measures ANOVA) for these variables (for participants who answered at all three time-points). As shown in figure 3, emotional exhaustion, job stress and emotion-focused coping changed mainly from baseline to one-year and not significantly from one- to three-year follow-up. All three subscales of job stress had a pattern similar to the main dimension of job stress. Neuroticism, however, trended to a significant decline from baseline to one year and changed significantly from one to three year follow-up. There were no significant changes in levels of active coping strategies.

### Panel modelling of the temporal relationships between change in emotional exhaustion and changes in job stress, coping and neuroticism

Cross-lagged panel models did not fit adequately or demonstrate significant cross lagged relationships between baseline and one- or three-year follow-up among emotional exhaustion, job stress, coping or neuroticism (not shown).

Synchronous panel models demonstrated several significant relationships, indicating that improvement in one variable was impacting change in the other variable in the model. These models fit well (Comparative Fit Index [CFI] range 0.98-1.00 and a standardized root mean square residual [SRMR] of 0.007 - 0.043, excepting social job stress and emotional exhaustion at three years that fit satisfactorily CFI 0.93, SRMR 0.073). Change in job stress with critical ratio CR (parameter/standard error distributed as z) = 3.16, p < 0.01 and emotion-focused coping with CR = 4.04, p < 0.001 preceded a reduction in emotional exhaustion at three-year follow-up. Similar relationships were found for one-year follow-up. When examining the three dimensions of job stress we found that social job stress had a similar pattern as total job stress. Change in emotional job stress impacted change in emotional exhaustion measured at one-year follow-up but not at three-year, whereas the temporal relationships between job stress due to fear of litigation and emotional exhaustion had different directions at one- and three-year follow-up. As expected, the relationship between active coping and emotional exhaustion was negative, indicating that more active coping was associated with reduction in emotional exhaustion. This relationship was significant at three-year follow-up, but not at one-year. Reduction in emotional exhaustion was found to occur sequentially before reduction in neuroticism both at one- and three-year follow-up (Table 2).

**Table 2:** Synchronous/co-temporal panel models of emotional exhaustion in relation to job stress (total and sub-scales), coping and neuroticism at one- and three-years follow-up

Synchronous Paths†	Baseline/1 year n = 149-152			Baseline/3 year n = 160-164		
	**Chi-square (df = 1)**	**Parameter/SE**	**CR‡**	**Chi-square (df = 1)**	**Parameter/SE**	**CR‡**

JS → EE	2.33	0.44/0.19	2.29**	6.63	0.72/0.23	3.16**
EE → JS		0.24/0.12	1.68		0.12/0.15	0.78

JS-Em → EE	0.68	0.36/0.16	2.32**	0.85	0.35/0.23	1.51
EE → JS-Em		0.10/0.11	0.91		0.13/0.12	1.05

JS-So → EE	3.67	0.43/0.14	3.12**	20.08	0.57/0.15	3.74***
EE → JS-So		0.15/0.19	0.76		0.16/0.20	0.78

JS-Fl → EE	0.25	0.10/0.16	0.65	0.14	0.49/0.19	2.62**
EE → JS-Fl		0.20/0.09	2.14**		0.05/0.12	0.43

AC → EE	2.43	-0.24/0.15	-1.63	0.50	-0.57/0.17	-3.32**
EE → AC		0.01/0.10	0.08		0.24/0.13	1.84

EC → EE	0.12	0.41/0.10	4.15***	1.19	0.48/0.12	4.05 ***
EE → EC		0.12/0.12	1.03		0.14/0.12	1.18

N → EE	4.19	0.09/0.06	1.50	0.98	0.09/0.07	1.38
EE → N		0.74/0.26	2.83**		0.70/0.23	3.11**

JS - Job Stress, EE - Emotional Exhaustion, JS-Em - Job Stress-Emotional dimension,

JS-So - Job Stress-Social dimension, JS-Fl - Job Stress-dimension of Fear of litigation.

AC - Active Coping, EC - Emotion-focused Coping, N - Neuroticism

†Confirmatory fit index range 0.93-1.00, Standardized root mean square residual 0.01-0.07

‡Critical ratio = parameter/standard error (distributed as z)

*p < 0.05, **p < 0.01, ***p < 0.001

### Role of psychotherapy

Physicians who received psychotherapy the first year after the intervention (n = 89) reported higher baseline levels of emotional exhaustion (3.17 SD 0.89 vs. 2.85 SD 0.97, p = 0.02) and neuroticism (2.91 SD 1.80 vs. 2.34 SD 1.78, p = 0.03) than the rest of the cohort (n = 95). However, there were no significant differences in coping strategies or job stress at baseline. The two groups had a similar overall change in emotion-focused coping over the three-year period. There was, however, a significant interaction between time and change in emotion-focused coping (F(2,318) = 4.54, p = 0.01). The repeated contrast indicated significant interactions both from baseline to one-year (F(1,159) = 4.96, p = 0.03) and from one- to three-year follow-up (F(1,159) = 8.90, p = 0.003). In repeated measures ANOVA for each group, the group in therapy showed some reduction in emotion-focused coping during the first year of follow-up (F(1,87) = 5.06, p = 0.03) but more change from one-year to three-year follow-up (F(1,87) = 8.94, p = 0.004). The group without therapy showed a reduction from baseline to one year (F(1,72) = 33.6, p < 0.001) but no further reduction from one to three years (F(1,72) = 0.67, ns). Levels at three-year follow-up were not significantly different between the groups. There were no interactions between therapy and time for reduction in emotional exhaustion, job stress, active coping or neuroticism.

## Discussion

In this prospective three-year follow-up study of physicians who sought a counselling intervention, we found that the significantly reduced level of emotional exhaustion, job stress and emotion-focused coping seen after one year, compared with baseline, were maintained at three-year follow-up [26]. The clinical significance of these results is indicated by the changes being of moderate effect sizes, describing a reduction in level of emotional exhaustion from significantly higher to a level not significantly different from Norwegian physicians in general, and a reduction in level of job stress from significantly higher to significantly lower than of Norwegian physicians in general [26]. Additionally, the enhanced work capacity, as indicated by a substantial reduction in the proportion of Sana physicians who were on sick leave at follow-up (both at one- and three-years) compared with baseline indicates a clinical significance.

The long-term reduction in emotional exhaustion contrasts with results from the few previous follow-up studies of preventive interventions for physicians that have found a reduction in emotional exhaustion up to a year after the intervention [25,30,40], but indicate a relapse without additional interventions [17,32]. In the present cohort there were no planned additional interventions. Some of the participants had, however, on their own initiative, chosen to come to a second intervention at the Resource Centre (primarily within the first six months, before the one-year follow-up), some had sought psychotherapy, and some had implemented a practical intervention by reducing weekly work hours, as reported previously [26]. All these post-intervention initiatives may have contributed to the reduction in emotional exhaustion over the years.

In this study there was a reduction in emotion-focused coping strategies occurring before the reduction in emotional exhaustion. Although previous studies have found that physicians under stress report more use of active coping strategies than their colleagues [11,12], this result indicates that it was a reduction in emotion-focused coping strategies, rather than an increase in active coping, that influenced reduction in emotional exhaustion. The reduction in emotion-focused coping, such as self-blame or wishful thinking, may have been a factor reducing the risk of relapse in emotional exhaustion in experience of renewed stress-exposure. The associations between active and emotion-focused coping strategies and their influence on emotional exhaustion warrant further investigation.

For the group of physicians attending psychotherapy after the counselling intervention a reduction in emotion-focused coping strategies was seen mainly from one- to three-year follow-up, whereas the group not attending psychotherapy showed a reduction from baseline to one-year follow-up. The former group was initially more distressed, reporting higher baseline levels of emotional exhaustion and neuroticism than the rest of the cohort. The results appear to indicate that some physicians can change their coping strategies after a short-term intervention (like the intervention at the Resource Centre, Villa Sana). The most distressed physicians, however, appeared to need additional psychotherapy that may have contributed to the significant reduction in emotion-focused coping found from one- to three-year follow-up in this group. These findings may strengthen previous recommendations of counselling and psychotherapy as primary and secondary preventive interventions for physicians [41,42]. This is in accord with studies of other distressed groups, where therapeutic interventions have been found to have the potential to change unfavourable coping strategies [13]. Since therapy in this study was self-selected, further work is needed to confirm this.

Longitudinal studies have previously shown reciprocal relationships between changes in emotional exhaustion and changes in job stress in normative samples of physicians [15,16]. In this study, however, a unilateral relationship was found, indicating that a reduction in perceived job stress occurred before reduction in emotional exhaustion. This may imply that the relationship between these parameters differs among physicians with high initial levels of emotional exhaustion, as in the present cohort, compared with physicians in normative samples.

As mentioned above, previous studies have shown a relationship between work-home interface stress and emotional exhaustion [2,4,16]. Consistent with this, we found that a reduction in the job stress dimension "social stress" (including both work-home interface stress and time pressure), preceded change in emotional exhaustion from baseline both to one- and three-year follow-up. The results for the two other job stress dimensions, emotional stress and stress due to fear of litigation were less consistent. Focusing on reduction in job stress, especially the social dimension may consequently be important in interventions for distressed physicians.

Neuroticism has previously been described as a relatively stable trait [28], whereas in this study there was a trend towards reduction from baseline to one year and significant reduction from one- to three-year follow-up. Neuroticism has previously been associated with, and has predicted, emotional exhaustion [3,8,28], whereas in this study changes in neuroticism (as estimated by the revised Eysenck's Personality Questionnaire) were preceded by reduction in emotional exhaustion. These relationships might differ between normative groups of physicians and a selected group, as in the present cohort. Further studies of the sequential relationships between changes in emotional exhaustion and personality traits in initially distressed doctors are needed.

### Strengths and limitations

A strength in this study is its prospective design with a three-year follow-up period and the relatively high proportion of participants completing one- and three-year follow-up (81%). Due to the study design, without a control group, we cannot determine whether the changes found are related to the intervention or whether they are a spontaneous regression towards the mean. A further possibility is that the changes could be related to factors not assessed in this study, for example social support. Our main objective, however, has been to investigate the three-year course of and the temporal relationships between the factors measured in this study after a counselling intervention

The five-point scoring scale of the Maslach Burnout Inventory (MBI) enabled us to compare the Villa Sana cohort with other Norwegian studies of physicians, but complicates direct comparisons with other international MBI-based studies, where a seven-point frequency scale is used. However, in this instance the former is more important than the latter, and regarding the relative roles of coping and job stress for change in emotional exhaustion we do not believe that the scale differences would have a substantive effect on the conclusions of the present study. Another limitation, reducing transnational generalizability of our results is the difference in working conditions for physicians in different countries. Although reduced work hours yield reduced emotional exhaustion both in Norway and in the U.S. [23-26], Norwegian physicians work fewer hours per week than e.g. American or British physicians [23-25] which could contribute to different relationship between the variables.

The sample size is important in structural equation modelling techniques [39,43]. In order to limit the number of parameters in the model, observed rather than latent variables were used. This was possible since the internal consistencies of the variables were acceptable. The constructed models fitted the data well, as indicated by the satisfactory model fit indices. In spite of restrictions due to the limited sample size, the main relationships between emotional exhaustion, job stress and emotion-focused coping strategies were generally consistent at one- and three-year follow-up.

This study indicates that reduction in emotion-focused coping and job stress preceded reduction in emotional exhaustion. These findings do not necessarily reflect the only possible lagged relationships among these parameters. As stated above, the intervention focused on the use of coping strategies and reduction of stress, and the temporal associations found might reflect this focus. Also, these changes are found in a group of physicians with initially elevated levels of emotional exhaustion who have decided to seek a counselling intervention and can therefore not be generalized to all physicians. Additionally, the literature indicates that there are other coping strategies like spirituality, not examined in this paper, which also could have importance for changes in emotional exhaustion.

Participants lost to three-year follow-up were more often men and had higher levels of distress (emotional exhaustion and job stress), as well as higher levels of emotion-focused coping strategies at baseline. It is difficult to estimate how inclusion of these participants would have influenced results concerning change of these parameters from baseline to follow-up as well as regarding how measures influence each other during follow-up. However, the proportion lost to follow-up was relatively small (19%).

## Conclusions

Follow-up of physicians who have participated in a counselling intervention showed that significantly reduced levels of emotional exhaustion, job stress, and emotion-focused coping found at one-year, compared with baseline, were maintained at three-year follow-up. The results of structural modelling were compatible with the assumption that reduction in emotion-focused coping and in job stress preceded reduction in emotional exhaustion. The clinical implication of these findings is that both change in coping strategies and reduction in job stress may be important foci for interventions with physicians aiming to reduce or prevent development of burnout.

## Competing interests

KR has been employed at the Resource centre Villa Sana, and is employed at the Research Institute Modum Bad. She has been reimbursed for presentation of preliminary results from the study at an internal meeting of the Norwegian Medical Association.

RT and TG run part-time, semi-private specialist practices in psychiatry that also include some physician-patients who have participated in the Villa Sana programmes.

TG, AH and HS are employed at the Research Institute, Modum Bad.

OA is employed by the Norwegian Medical Association.

There are no other competing interests.

## Authors' contributions

KR and TG conceptualized and designed the study, developed the construction of the questionnaire, analyzed and interpreted data and drafted the paper. RT also contributed to drafting the paper. AH and HS have contributed to analyzing and interpreting data, while OA has participated in the development and the construction of the questionnaire.

All authors have participated in revising the manuscript critically for important intellectual content and have approved the final manuscript.

## Pre-publication history

The pre-publication history for this paper can be accessed here:

http://www.biomedcentral.com/1471-2458/10/213/prepub

## Supplementary Material

Additional file 1 **An abbreviated version of Vitaliano's Ways of coping check list**. The items from Vitaliano's Ways of coping check list that are used in the present study. Click here for file
