# Supplementary material for: A three-year cohort study of the relationships between coping, job stress and burnout after a counselling intervention for help-seeking physicians
Source: BMC Public Health. 2010 Apr 27;10:213. doi: 10.1186/1471-2458-10-213 (PMC2880293; doi:10.1186/1471-2458-10-213)
Supplement: Additional file 1 — An abbreviated version of Vitaliano's Ways of coping check list. The items from Vitaliano's Ways of coping check list that are used in the present study. [file 1471-2458-10-213-S1.DOC]

Additional file 1:

**An abbreviated version of Vitaliano´s Ways of coping check list.
The items from Vitaliano´s Ways of coping check list that are used in the present study.**

1. Changed or grew as a person in a positive way. AC
2. Wished the situation would go away or somehow be finished. EC
3. Talked to someone who could do something about the problem AC
4. Wished I could change the way that I felt. EC
5. Wished I was a stronger person – more optimistic and forceful. EC
6. Talked to someone to find out about the situation. AC
7. Day-dreamed or imagined a better time or place than the one I was in. EC
8. Came up with a couple of different solutions to the problem. AC
9. Blamed yourself. EC
10. Talked to someone about how I was feeling. AC
11. Wished that I could change what had happened. EC
12. Asked someone I respected for advice and followed it. AC
13. Came out of the experience better than when I went in. AC
14. Thought about fantastic or unreal things (like perfect revenge or EC
    finding a million dollars) that made me feel better.
15. Changed something so things would turn out all right. AC

1. Had fantasies or wishes about how things might turn out. EC
2. Accepted sympathy and understanding from someone. AC
3. Criticized or lectured yourself. EC

AC – statement describing Active Coping
EC – statement describing Emotion-focused Coping
